# Control of growth and inflammatory response of macrophages and foam cells with nanotopography

**DOI:** 10.1186/1556-276X-7-394

**Published:** 2012-07-16

**Authors:** Mohammed Mohiuddin, Hsu-An Pan, Yao-Ching Hung, Guewha Steven Huang

**Affiliations:** 1 Department of Material Science and Engineering, National Chiao Tung University, 1001 University Road, Hsinchu 300, Taiwan; 2 Section of Gynecologic Oncology, Department of Obstetrics and Gynecology, China Medical University and Hospital, 91 Hsueh Shih Road, Taichung 404, Taiwan; 3 College of Medicine, China Medical University, Taichung 404, Taiwan

**Keywords:** Cell adhesion, Nanotopography, Macrophages, Foam cell, Biocompatible, Inflammatory response

## Abstract

Macrophages play an important role in modulating the immune function of the human body, while foam cells differentiated from macrophages with subsequent fatty streak formation play a key role in atherosclerosis. We hypothesized that nanotopography modulates the behavior and function of macrophages and foam cells without bioactive agent. In the present study, nanodot arrays ranging from 10‐ to 200‐nm were used to evaluate the growth and function of macrophages and foam cells. In the quantitative analysis, the cell adhesion area in macrophages increased with 10- to 50-nm nanodot arrays compared to the flat surface, while it decreased with 100- and 200-nm nanodot arrays. A similar trend of adhesion was observed in foam cells. Immunostaining, specific to vinculin and actin filaments, indicated that a 50-nm surface promoted cell adhesion and cytoskeleton organization. On the contrary, 200-nm surfaces hindered cell adhesion and cytoskeleton organization. Further, based on quantitative real-time polymerase chain reaction data, expression of inflammatory genes was upregulated for the 100- and 200-nm surfaces in macrophages and foam cells. This suggests that nanodots of 100‐ and 200‐nm triggered immune inflammatory stress response. In summary, nanotopography controls cell morphology, adhesions, and proliferation. By adjusting the nanodot diameter, we could modulate the growth and expression of function-related genes in the macrophages and foam cell system. The nanotopography-mediated control of cell growth and morphology provides potential insight for designing cardiovascular implants.

## Background

Recent fabrication of nanostructured materials with different surface properties has generated a great deal of interest for developing implant materials, i.e., cardiovascular, dental, orthopedic, percutaneous, subcutaneous, and auditory [1-5]. The interface between nanostructured materials and biological tissues is likely to vary dependent upon the surface properties of the nanomaterial. Understanding the degree of toxicity induced by the unique cellular interaction of nanostructured materials is a major concern before utilization in biomedical applications [6-8]. Therefore, fabricating biocompatible materials which are designed to perform specific functions within living organisms has become a key component for generating nanodevices for biomedical applications, including implants.

Macrophages play a critical role during innate and acquired immune responses through the phagocytosis of foreign material. During an immune response, macrophages are typically the first cell type to respond and will secrete proteins (cytokines and chemokines) in order to recruit more immune cells to the site of injury. Atherosclerosis is a pathological process that takes place in the major arteries and is the underlying cause of heart attacks, stroke, and peripheral artery disease. The earliest detectable lesions, called fatty streaks, contain macrophage foam cells that are derived from recruited monocytes. The formation of these foam cells correlates to inflammatory responses [9-11]. In particular, immune cells such as monocytes and macrophages play a key role in mediating host tissue response to implants in the foreign body reaction.One study demonstrated that the macrophage receptor with collagenous structure (MARCO) displayed limited expression in healthy cells but increased in expression around the synovial fluid following hip replacements [12]. This study indicated that the presence of a foreign body can generate an immune response, and the continued presence of the foreign body can potentially lead to macrophage buildup and production of foam cells.

Recent reports have shown that microscaled landscapes are able to direct shape and migration of cultured cells. When cultured on ridges and grooves of nanoscale dimensions, cells migrate more extensively to the ridges than into the grooves. Cell shape is aligned and extended in the direction of the groove [13]. Osteoblasts grown on a fibrous matrix composed of multiwalled carbon nanofibers (100-nm in diameter) exhibit increased proliferation compared to those on flat glass surfaces [14-16]. Nanodots larger than 100-nm in diameter induced an apoptosis-like morphology for NIH-3T3 fibroblast cells [17]. Breast epithelial cells proliferate and form multicellular spheroids on interwoven polyamide fibers fabricated using electrospinning polymer solution onto a glass slide [18]. A 3-D nanofibrillar surface covalently modified with tenascin-C-derived peptides enhances neuronal growth *in vitro*[19]. The cardiomyoblast H9c2 shows induced cell adhesion and cytoskeleton organization on nanodot arrays smaller than 50-nm [20].

Recently, arrays of nanodots with defined diameter and depth have been fabricated using aluminum nanopores as a template during oxidation of tantalum thin films [21]. The pore size of aluminum oxide is controllable and uniformly distributed; the depth of dots depends on the voltage applied; thus, it can serve as a convenient mold to fabricate tantalum into a nanodot array of specific diameter and depth. The structure containing nanodots of uniform size could serve as a comparable nanolandscape to probe cellular response at the molecular level. Although many implant surface topographies are commercially available, there is generally a lack of detailed comparative histological studies at the nano-interface that document how these surfaces interact with living cells, in particular immune cells. In the present study, different sizes of nanodot arrays ranging from 10- to 200-nm were used to evaluate the growth and inflammatory response of macrophages and foam cells.

## Methods

### Chemicals

Dulbecco’s modified Eagle medium (DMEM), FBS, antibiotics, and all other tissue culture reagents were obtained from Gibco (Life Technologies, Carlsbad, CA, USA). Glutaraldehyde and osmium tetroxide were purchased from Electron Microscopy Sciences (Hatfield, PA, USA). Anti-vinculin mouse antibody was purchased from Abcam (Cambridge, MA, USA). Alexa Fluor 594 phalloidin and Alexa Fluor 488 goat anti-mouse IgG were purchased from Invitrogen (Carlsbad, CA, USA). Trypsin was purchased from Sigma-Aldrich (St. Louis, MO, USA). CuSO4, KBr, thiobarbituric acid, trichloroacetic acid, and other commonly used chemicals were purchased from Sigma-Aldrich or Merck (Whitehouse Station, NJ, USA).

### Isolation of mouse peritoneal macrophages and formation of foam cells

Resident peritoneal macrophages were isolated and cultured from five mice (20 g each) and were washed once with DMEM and once with DMEM containing 10% fetal bovine serum. The cells were seeded on to different nanodot arrays, ranging from 10- to 200-nm, in DMEM containing 10% fetal bovine serum and 100 pg/ml penicillin and cultured for 3 to 4 h at 37°C in an incubator containing 5% CO_2_ with 90% humidity. The nonadherent cells were removed, and the monolayers were then placed in DMEM containing 10% fetal bovine serum supplemented with 100 μg/ml oxidized low-density lipoprotein (ox-LDL) or acetyl LDL; plates were further incubated for an additional 72 h.

### Oil red O staining

Monolayers of foam cells prepared on nanodot surface were fixed with 10% formaldehyde in phosphate-buffered saline (PBS) (pH 7.4) for 10 min at room temperature, then stained with Oil Red O, and counterstained with hematoxylin for 10 min [22].

### Fabrication and characterization of nanodot arrays

Nanodot arrays were fabricated by anode aluminum oxide processing as described previously [21]. A tantalum nitride (TaN) thin film of 150-nm thickness was sputtered onto a 6-in. silicon wafer followed by deposition of a 3-μm-thick aluminum onto the top of a TaN layer. Anodization was carried out in 1.8 M sulfuric acid at 5 V for the 10-nm nanodot array or in 0.3 M oxalic acid at 25, 60, and 100 V for 50-, 100-, and 200-nm nanodot arrays, respectively. Porous anodic alumina was formed during the anodic oxidation. The underlying TaN layer was oxidized into tantalum oxide nanodots using the alumina nanopores as a template. The porous alumina was removed by immersing in 5% (*w*/*v*) H_3_PO_4_ overnight.

The dot diameters were 15 ± 2.8-nm, 58.1 ± 5.6-nm, 95.4 ± 9.2-nm, and 211.5 ± 30.6-nm for the 10-, 50-, 100-, and 200-nm dot arrays. The average heights were 11.3 ± 2.5-nm, 51.3 ± 5.5-nm, 101.1 ± 10.3-nm, and 154.2 ± 27.8-nm, respectively. Dot-to-dot distances were 22.8 ± 4.6-nm, 61.3 ± 6.4-nm, 108.1 ± 2.3-nm, and 194.2 ± 15.1-nm, respectively. The dimension and homogeneity of nanodot arrays were measured and calculated from images taken using a JEOL JSM-6500 TFE-SEM (JEOL Ltd., Akishima, Tokyo, Japan).

### The cell viability assay

Cells were harvested and fixed with 4% formaldehyde in PBS for 30 min followed by PBS wash for three times. The membrane was permeated by incubating in 0.1% Triton X-100 for 10 min, followed by PBS wash for three times. The sample was incubated with 4′,6-diamidino-2-phenylindole (DAPI) and phalloidin for 15 min at room temperature. The samples were mounted and imaged using a Leica TSC SP2 confocal microscope (Leica Microsystems Ltd., Milton Keynes, UK). The number of viable cells was counted using ImageJ software (National Institutes of Health, Bethesda, MA, USA) and expressed in terms of cell density.

### Scanning electron microscopy

The harvested cells were fixed with 1% glutaraldehyde in PBS at 4°C for 20 min, followed by post-fixation in 1% osmium tetroxide for 30 min. Dehydration was performed through a series of ethanol concentrations (10-min incubation each in 50%, 60%, 70%, 80%, 90%, 95%, and 100% ethanol) and air-dried. The specimen was sputter-coated with platinum and examined using a JEOL JSM-6500 TFE-SEM at an accelerating voltage of 5 keV.

### Immunostaining

Cells were harvested and fixed with 4% paraformaldehyde in PBS for 15 min followed by PBS wash for three times. Cell membrane was permeated by incubating in 0.1% Triton X-100 for 10 min, followed by PBS wash for three times, and blocked by 1% bovine serum albumin (BSA) in PBS for 1 h and PBS wash for three times. The sample was incubated with anti-vinculin antibody (properly diluted in 1% BSA) and phalloidin for 1 h, followed by incubation with Alexa Fluor 488 goat anti-mouse antibody for 1 h, and then followed by PBS wash for three times. Samples were mounted and imaged by using a Leica TSC SP2 confocal microscope.

### Quantitative real-time RT-PCR

Total RNA was extracted from macrophages and foam cells using TRI-reagent (Talron Biotech, Rehovot, Israel) according to the manufacturer’s specifications. The RNA was isolated using chloroform extraction and isopropanol precipitation. The crude RNA extract was immediately purified with an RNeasy Mini Kit (Qiagen, Venlo, Netherlands) to remove impurities and unwanted organics. Purified RNA was resuspended in DEPC water and quantified by OD_260_. The OD_260_ to OD_280_ ratio usually exceeded 2.0 at this stage. For cDNA synthesis, 1 μg of total RNA was annealed with 1 μg of oligo-dT primer, followed by reverse transcription using Super Script® III Reverse Transcriptase (Invitrogen, Carlsbad, CA, USA) in a total volume of 50 μl. Between 0.2 and 0.5 μl of the reverse transcription reactions was used for quantitative polymerase chain reaction (qPCR) using SYBR Green I on an iCycler iQ5 (Bio-Rad Laboratories, Hercules, CA, USA). Cycling conditions were as follows: 1× (5 min at 95°C) and 50× (20 s at 95°C, 20 s at 55°C, and 40 s at 72°C). Fluorescence was measured after each 72°C step. Expression levels were obtained as threshold cycles (Ct), which were determined by the iCycler iQ Detection System software (Bio-Rad Laboratories, Hercules, CA, USA). Relative transcript quantities were calculated using the ΔΔCt method. Glyceraldehydes-3-phosphate dehydrogenase (GAPDH) was used as a reference gene and was amplified from the same cDNA samples. Due to the difference in threshold cycles of the sample mRNA relative to the GAPDH, mRNA was defined as ΔCt. The difference between the ΔCt of the untreated control and the ΔCt of the SMF-treated sample was defined as ΔΔCt. The fold change in mRNA expression was expressed as 2^ΔΔCt^. The results were expressed as the mean ± standard deviation (SD) of six experiments.

## Results and discussion

### Nanotopography-modulated morphology and cell spread of macrophages and foam cells

**Figure 1:**
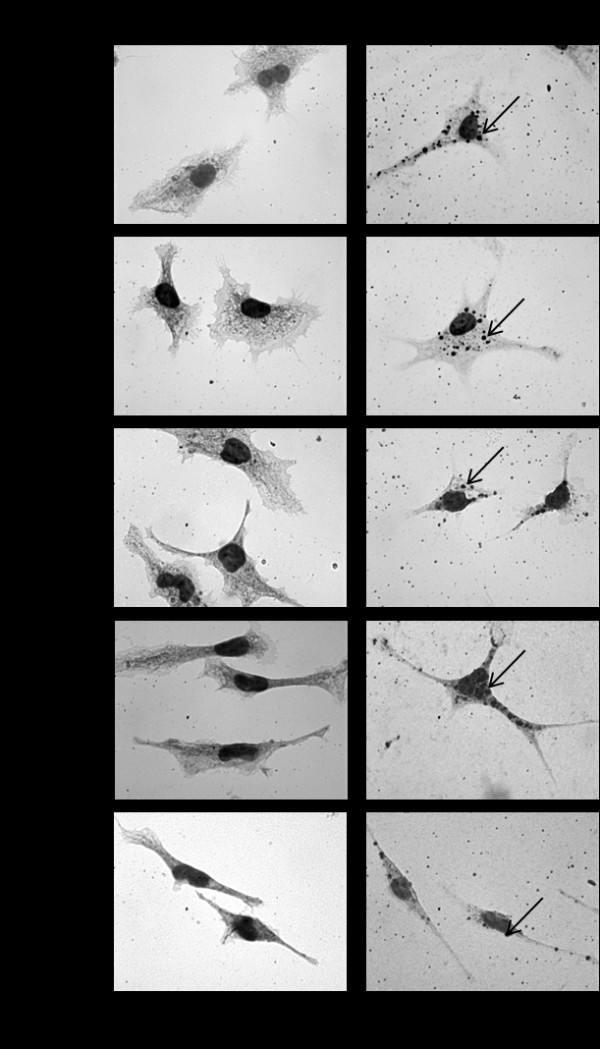
**Macrophage and foam cell morphology changes with topographical surface.** Macrophages and foam cells were grown on flat, 10-, 50-, 100-, and 200-nm nanodot arrays for 3 days, stained with Oil Red O, and counter stained by hematoxylin (the arrows in foam cells indicate engulfed lipoproteins). Morphology was imaged by optical microscopy. Scale bar = 50 μm.

**Figure 2:**
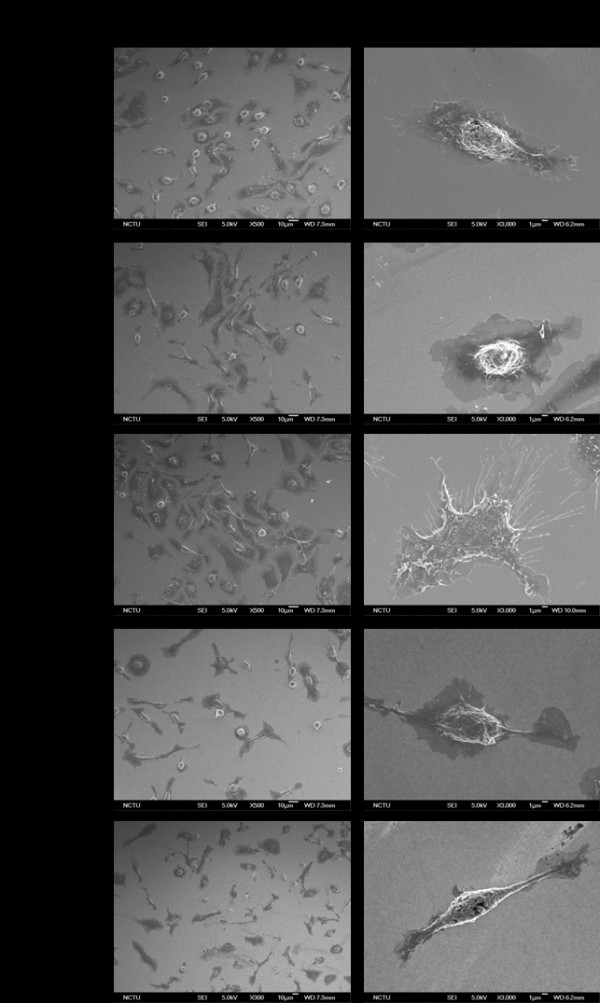
**Morphology of macrophages cultured on nanodot arrays.** Macrophages were grown on flat, 10-, 50-, 100-, and 200-nm nanodot arrays for 3 days, and their morphology was imaged by scanning electron microscopy. Scale bar for low-mag = 10 μm and for high-mag = 1 μm.

To characterize how the macrophages and foam cells interact with the aforementioned nanodot arrays, the cells were cultured for 72 h on nanodot arrays of different diameters including flat substrate as a control. The morphological appearance of adhered cells was imaged by both optical image microscopy and scanning electron microscopy (SEM) (Figures 1 and 2). Macrophages and foam cells grown on the flat surface and 10-nm nanodot arrays exhibited flat and extended conformation during the course of 3 days. Cells grown on the 50-nm nanodot arrays showed more extended morphology than those on the flat surface with apparently larger surface area for each cell. Cells grown on the 100-nm nanodot arrays exhibited a distorted morphology with shrinking surface area and increased length of lamellipodia. Apoptosis-like appearance and reduction in surface area with extended lamellipodia were seen with cells seeded on the 200-nm nanodot arrays.

**Figure 3:**
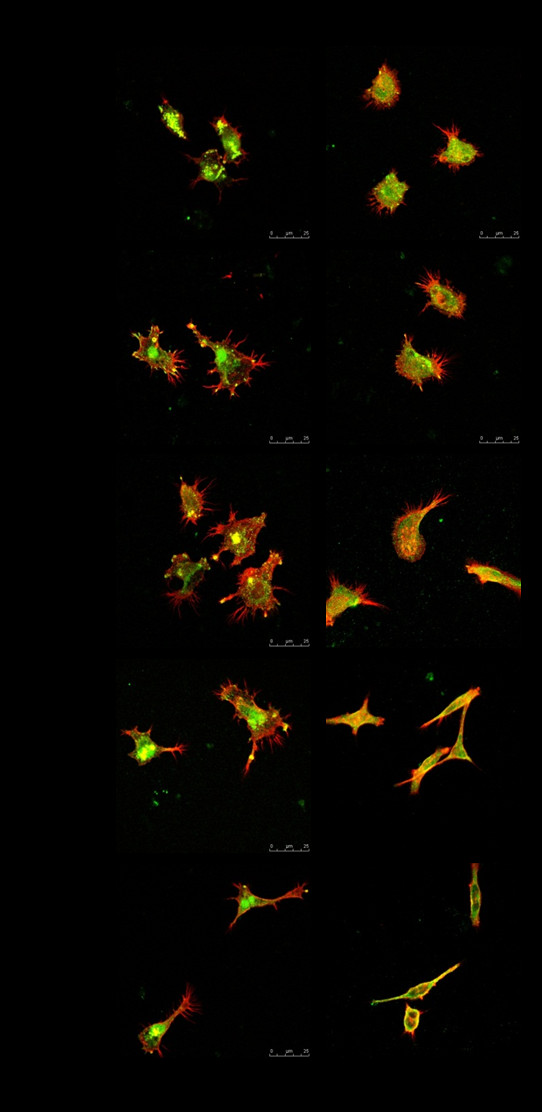
**Nanotopography-dependent cell spreading area, focal adhesion, and cell density.** Macrophages and foam cells seeded on nanodot arrays of various sizes were harvested after a 3-day culture. (**a**) Cell spread area versus dot diameter for cells cultured on the nanodot arrays. The viable cells were counted, and the percent adhesion area relative to cells cultured on a flat surface was calculated and plotted against the nanodot diameter. (**b**) Focal adhesions (amount of vinculin staining) versus dot diameters for cells cultured on nanodot arrays. The amount of vinculin staining per cell was measured, and the percentage of focal adhesion relative to cells cultured on a flat surface was calculated and plotted against the nanodot diameter. (**c**) Cell density versus dot diameter for cells cultured on the nanodot arrays. The viable cells were counted, and percent viability relative to cells cultured on a flat surface was calculated and plotted against the nanodot diameter. The mean ± SD from at least three experiments is shown. Asterisk denotes *p* < 0.005 when compared to the flat control surface.

Formation of focal adhesions reflected by the attachment of filopodia and lamellipodia to the substratum indicates healthy growth for cultured cells. SEM images showed that the lamellar body of migrating cells, seeded on 50-nm nanodot arrays, exhibited wide and thick characters with a large number of filopodia (Figure 2). Cells seeded on flat and 10-nm nanodot arrays showed comparable lamellipodia. However, the cells seeded on 100- and 200-nm nanodot arrays were mounted with extended length and narrow-size lamellipodia. Figure 3a shows the correlation between cell spread area and dot size for macrophages and foam cells . Cell surface area representing the percent adhesion area of viable cells relative to cells cultured on a flat surface was calculated and plotted against the nanodot diameter. Based on quantitative analysis, the cell spread area of macrophages increased significantly between 21.6% and 37.9% with increasing dot sizes of 10- to 50-nm, respectively. Interestingly, as dot diameter increased from 100- to 200-nm, there was a significant reduction in cell surface area of 22.7% and 43.2%, respectively. A similar biphasic trend, increasing cell surface area from 10- to 50-nm and decreasing from 100- to 200-nm, was observed with foam cells. This trend of change correlated with qualitative analysis as shown in Figure 1. Although the precise reason for such differential growth pattern is not known, since increasing the dot diameter above 100-nm stimulates an apoptosis-like growth and a significant reduction in the surface area, these results demonstrate that nanodot arrays larger than 100-nm are less biocompatible to cells.

### Nanotopography-modulated cell adhesion and cytoskeleton organization of macrophages and foam cells

**Figure 4:**
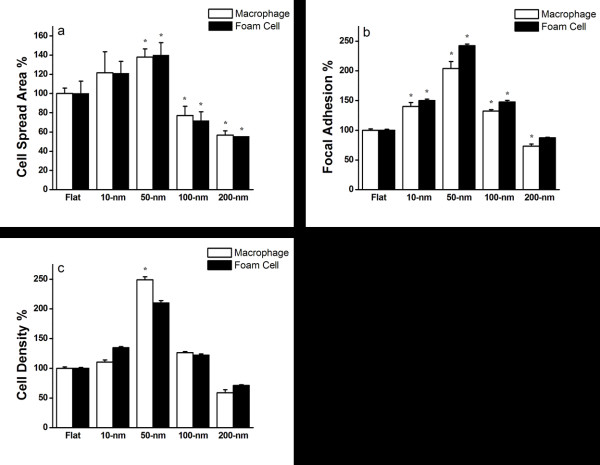
**Immunostaining showing distribution of vinculin (green) and actin filaments (red) in macrophages and foam cells.** Macrophages and foam cells were seeded on flat, 10-, 50-, 100-, and 200-nm nanodot arrays for 3 days, and their morphology was observed by confocal microscopy. Scale bar = 25 μm.

To evaluate cell adhesion and cytoskeleton reorganization, immunostaining specific to vinculin and actin filaments was performed on nanodot arrays (Figure 4). The amount of vinculin staining in foam cells was significantly less than that in macrophages for all nanodot sizes. Foam cells had fewer focal adhesion molecules than macrophages when grown on nanodot arrays and might have the destiny of apoptosis. Vinculin staining was well distributed for macrophages grown on the flat surface and on the 10- to 100-nm nanodot arrays, with the highest density of vinculin for cells grown on 50-nm nanodot arrays. Nevertheless, the amount of vinculin staining decreased for cells grown on 200-nm nanodot arrays (Figure 4). For foam cells, vinculin staining had the same trend as that for macrophages: increasing from flat to 50-nm nanodot arrays, becoming gradually lost for cells grown on 100-nm nanodot arrays, and completely disappearing for 200-nm nanodot arrays.

Immunostaining of actin filaments indicated a well-organized cytoskeleton in macrophages grown on flat, 10-, and 50-nm nanodot arrays, but it is gradually lost for 100-nm nanodot arrays and has completely disappeared for 200-nm nanodot arrays. For foam cells, cytoskeleton arrangement had the same trend as macrophages: increasing from flat to 50-nm nanodot arrays, becoming gradually lost for cells grown on 100-nm nanodot arrays, and completely disappearing for 200-nm nanodot arrays.

Immunostaining indicated that nanodot arrays in the range of 10- to 100-nm promoted cell adhesion and cytoskeleton organization for macrophages and foam cells (Figure 3b). Best adhesion occurred at 50-nm nanodots, whereas nanodots of 200-nm retarded the formation of focal adhesions and inhibited the organization of the cytoskeleton.

### Nanotopography-modulated cell density

To evaluate the viability of macrophages and foam cells on varied nanodot arrays, cells were seeded on nanodot arrays, ranging from 10- to 200-nm including flat control. Macrophages and foam cells were cultured for 72 h, and then, DAPI staining was performed to verify viable cells on each nanodot array and flat surface (Figure 3c). For macrophages, compared to the flat surface, there were 10.6%, 149.1%, and 26.5% increases in the number of viable cells for 10-, 50-, and 100-nm nanodot arrays, respectively, but 41.2% reduction was observed on 200-nm nanodot arrays. For foam cells, a 110% increase in the number of viable cells was observed for 50-nm nanodot arrays, and 28.6% reduction occurred for 200-nm nanodot arrays.

### Effect of nanotopography on the expression of genes related to inflammation and circulatory repair

**Figure 5:**
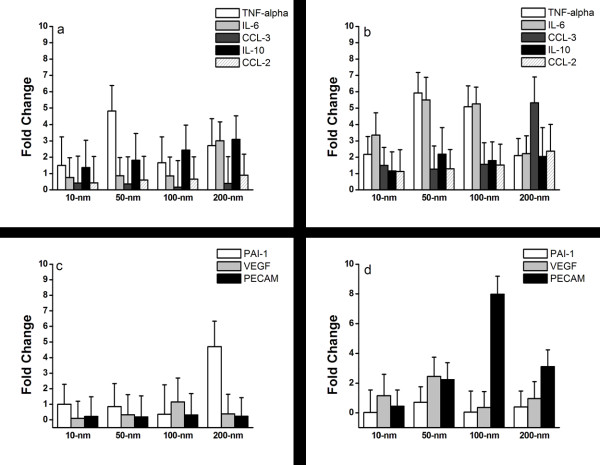
**Changes in cytokine and chemokine gene expression profiles.** (**a**) Macrophages and (**b**) foam cells were cultured on nanodot arrays of different sizes for 72 h, and qPCR was conducted to evaluate gene expression of TNF-α, IL-6, CCL-3, IL-10, and CCL-2. Gene expression profiles for genes were involved in circulatory repair. (**c**) Macrophages and (**d**) foam cells were cultured on nanodot arrays of different sizes for 72 h, and qPCR was conducted to evaluate gene expression of PAI-1, VEGF and PECAM.

The cytokine gene expression profiles related to inflammation of macrophages and foam cells cultured on nanodot arrays were measured at 72 h, using qPCR. The cytokines examined were TNF-α, IL-6, IL-10, CCL-2, and CCL-3 (Figure 5a,b). In addition, genes important for the development of the circulatory system and repair were also evaluated (Figure 5c,d): PAI-1, VEGF, and PECAM.

TNF-α is a pro-inflammatory cytokine and is responsible for activation and positive regulation of the NFκB pathway, which is a key regulator of the immune response [23]. There was a five-fold increase in TNF-α found at 50-nm nanodot arrays in macrophages, while a five- to six-fold increase was demonstrated in foam cells at 50- to 100-nm nanodot arrays (Figure 5a,b). Similarly, IL-6 encodes a pro-inflammatory cytokine that is critical for activating an acute inflammatory response and is responsible for recruiting adaptive immune cells to the site of injury or infection. There was a significant three-fold increase of IL-6 in macrophages cultured on 200-nm nanodot arrays, while there was about a one-fold increase at 50- to 100-nm nanodot arrays. In contrast, the foam cells responded differently than the macrophages, with a three- to five-fold increase of IL-6 when cultured on 10- to 100-nm nanodot arrays and a two-fold increase for 200-nm nanodot arrays. In comparison, IL-10 is an anti-inflammatory cytokine responsible for blocking the synthesis of pro-inflammatory cytokines and negatively regulates NFκB activation. There was an increasing trend of IL-10 gene expression in the macrophages and the foam cells (Figure 5a,b). For the CCL-2 chemokine, which functions to recruit monocytes and other immune cells, there was no significant change in any of the macrophage topography conditions. However, in foam cells, there was a 2.5-fold increase at 200-nm nanodot arrays, while there was less than one-fold increase at 10- to 100-nm nanodot arrays (Figure 5b). Another common chemokine is CCL-3. This protein is responsible for recruiting and activating leukocytes to aid in an immune response. Figure 5a showed less than 1% increase in CCL-3 in macrophages at 10- to 200-nm nanodot arrays, while a six-fold increase at 200-nm nanodot arrays in foam cells was observed (Figure 5b).

In addition to the immune response, genes associated with circulatory repair were also investigated. PAI-1 is a gene involved in the prevention of blood clots. There was a five-fold increase in PAI-1 observed at 200-nm nanodot arrays in macrophages (Figure 5c), whereas foam cells displayed an inconsistent pattern of increase of PAI-1 (Figure 5d). Furthermore, VEGF is known to play a key role in the creation of new blood vessels during development as well as repair during injury. There was an inconsistent increase in VEGF for the macrophages, while foam cells demonstrated a significant increase at the 50-nm topography (Figure 5d). PECAM is responsible for removing old neutrophils from the site of injury and preventing buildup of immune cell debris. In the macrophages, there was no change in PECAM genes; however, the foam cells displayed significant increases for the 50- to 200-nm topographies. In summary, the differences in relative gene expressions shown here are topography induced and normalized to flat surface.

When evaluating the gene expression trends observed in the macrophages, there was a highly significant increase in TNF-α expression for the 50-nm topography. For all of the topographies, there was limited impact on the expression of CCL-3 and CCL-2, whereas, there was a mild elevation in IL-6 expression. Taken together, there was a limited induction of an inflammatory response for these topographies. The expression of PAI-1, VEGF, and PECAM were also evaluated, and there were low levels of expression for all genes in all topographies except for the 200-nm topography, which displayed significant increases in PAI-1 expression. Since PAI-1 helps to prevent blood clots and the buildup of cells, in conjunction with data from the gene expression studies where limited immune responses were observed, the 200-nm topography might assist in the prevention of foam cell formation.

When evaluating the foam cells directly, there were high levels of the early-response pro-inflammatory cytokines IL-6 and TNF-α for the 50- and 100-nm topographies with mild increases in expression for the 10- and 200-nm topographies. Furthermore, the 200-nm topography demonstrated high levels of expression for the pro-inflammatory cytokine CCL-3. In addition, when the circulatory repair genes were assessed, the 100-nm topography demonstrated a significant increase in the PECAM gene which functions to remove leukocyte debris from cells. Since foam cells are derived from macrophages that are not cleared from the system, this 100-nm topography could greatly aid in the prevention or removal of foam cells from the body.

After 72 h, macrophages showed an increase in inflammatory gene expression on 10- and 200-nm nanodot arrays, while the difference was not significant compared to the flat. Foam cells showed the most inflammatory gene expression on 200-nm nanodot arrays. The common acute inflammation gene expression of CCL-3 responded significantly to the topography of 100- and 200-nm nanodot arrays for foam cells. However, the macrophages showed the most acute inflammation on the 10-nm nanodot arrays. Thus, the topographical effect on the PAI-1 gene expression was difficult to discern.

Recently, it has been shown that three-dimensional surface topography (size, shape, and surface texture) is one of the most important parameters that influence cellular reactions [24,25]. Other studies have demonstrated that the difference in cellular response correlates with a modulation of the concentration of serum proteins on the surface [26,27]. Many studies have shown that cell biomaterial interactions can activate macrophages which results in the synthesis of pro-inflammatory agents such as TNFα, IFNγ, IL-1, and IL-6 [28,29] Most likely, the surface properties, such as material surface chemistry and topography, can modulate the expression of pro-inflammatory cytokines and chemokines by macrophages in a time-dependent manner [30].

Although many studies have investigated cellular reaction to different surface patterns, the behavior of immune cells, such as macrophages and foam cells, cultured on different diameters of nanodots has not been studied thoroughly. Our study suggests that topography may modulate the phenotypes of macrophages and foam cells in the context of foreign body response. The response to topography in the form of nanodot arrays in the range of 10- to 200-nm has revealed a distinctive pattern, and topography indeed affects cell morphology, density, adhesion, and cytokine expression compared to flat controls. The changes in cell morphology are observed in four different sizes of nanodot arrays, indicating that the findings in this study are topography-mediated. Using topography-induced change in macrophages and foam cell behavior, it is possible to influence phenotypic response, such as cell activation, motility, and maturation in the foreign body response. While there is a mild induction of the inflammatory response for the 100- and 200-nm topographies, these growth conditions also supported expressions of genes that would be responsible in the prevention of foam cell formation and the removal of foam cells, suggesting potential benefits. Furthermore, it might be possible to treat the cells with antioxidants or other anti-inflammatory mediators to prevent the inflammatory response while benefiting from the increase in PAI-1 and PECAM.

## Conclusion

We have shown topologic modulation of cell growth, cell density, cell spreading area, and immune functions. Our results demonstrated that 50-nm nanodots displayed a biocompatible surface compared to 100- and 200-nm nanodots in terms of macrophage and foam cell growth. In addition, based on qPCR data, 100- and 200-nm surface-induced inflammatory gene expression in macrophages and foam cells suggest that nanostructured materials (100- and 200-nm) trigger the immune inflammatory stress response. The role of topography in modulating implant tissue reaction would require further elucidation. This study suggests that nanotopography may be beneficial for the design of cardiovascular implants.

## Competing interests

The authors declare that they have no competing interests.

## Authors’ contributions

MM and HAP are responsible for concept and design. MM carried out the experiments, analyzed the data, and wrote the paper under the close supervision of YCH and GSH. All authors read and approved the final manuscript.

## Authors’ information

MM and HAP are doctoral degree students at the Department of Material Science and Engineering, National Chiao Tung University. GSH is a professor at the Department of Material Science and Engineering, National Chiao Tung University. YCH is a professor at the Department of Obstetrics and Gynecology, China Medical University and Hospital and also professor at the College of Medicine, China Medical University.
